# Phytochemical Investigations and Pharmacological Potential of Organic Extracts of *Calotropis gigantea* L. Leaves

**DOI:** 10.1155/sci5/1669969

**Published:** 2025-07-08

**Authors:** Adil Jamal, Amina Arif, Shumaila Kiran, Muhammad Naveed Shahid, Md. Belal Hossain

**Affiliations:** ^1^Department of Biochemistry and Biotechnology, Faculty of Sciences, The University of Faisalabad, Faisalabad 38000, Punjab, Pakistan; ^2^Sciences and Research, College of Nursing, Umm Al-Qura University, Makkah 715, Saudi Arabia; ^3^Faculty of Science and Technology, University of Central Punjab, Lahore 54000, Pakistan; ^4^Department of Applied Chemistry, Government College University, Faisalabad 38000, Pakistan; ^5^Department of Botany, Division of Science and Technology, University of Education, Lahore 54770, Pakistan; ^6^Department of Plant Pathology, Faculty of Agriculture, Sher-e-Bangla Agricultural University, Sher-e-Bangla Nagar, Dhaka 1207, Bangladesh

**Keywords:** antimicrobial, antioxidant, *C. gigantea*, DPPH, flavonoids, hemolytic, phenolic

## Abstract

*Calotropis gigantea* holds significant therapeutic value in Indian traditional medicine, since it is utilized for the treatment of several diseases. The current study was performed to evaluate the phytochemical profile, antioxidant radical scavenging (DPPH), and hemolytic and antimicrobial activities of organic fractions of *C. gigantea* leaves. Qualitative and quantitative analysis of phytochemicals, antioxidant components, phenolics, and flavonoids were conducted using organic solvents, including methanol, n-hexane, chloroform, ethyl acetate, n-butanol, and aqueous solutions. The bioactive fractions extracted from the leaf were tested against pathogenic organisms (*Escherichia coli, Pseudomonas aeruginosa, Enterococcus faecalis, Aspergillus fumigatus, Aspergillus flavus*, and *Aspergillus niger*) using the agar well diffusion and microdilution broth method. Hemolytic assay was employed to measure erythrocyte damage in response to bioactive fractions. Our study reflected the presence of bioactive constituents (alkaloids, saponins, tannins, flavonoids, terpenoids, cardiac glycosides, and quinones) with abundance of these found in all except *n*-hexane, ethyl acetate, and aqueous. All extracts contained significant amounts of total phenolics and total flavonoids (μg/mg). DPPH activity measured was maximum in methanolic aqueous followed by *n*-hexane, ethyl acetate, chloroform, and *n*-butanol. Methanol, ethyl acetate, and aqueous fractions showed maximum inhibition antibacterial activity in all Gram-positive and Gram-negative strains while antifungal activity tested in all fungal strains against all fractions showed promising inhibition. The results of our investigation indicate that the organic fractions exhibited reduced hemolytic activity, suggesting an enhanced medicinal potential and decreased toxicity of *C. gigantea* extracts. Based on the current results, it can be inferred that extracts derived from *C. gigantea* have diverse potential and urge further investigation as a possible reservoir of metabolites, antioxidants, and antibacterial compounds. Nevertheless, it is imperative to utilize an appropriate and standardized solvent extraction methodology in order to recover possible phytochemicals and antioxidants that possess therapeutic properties from *C. gigantea*.

## 1. Introduction

Medicinal plants have been valuable source of human health since ancient ages. Plants have made significant contributions to the provision of drugs, clothes, shelter, and food in a particular manner. Extensive research has been conducted on natural compounds to identify new drugs [1]. Certainly, plants have been employed for more than centuries [2] as source of analgesics, antibiotics, anticancerous, and cardioprotective, among others [3]. In developing countries, approximately 70%–90% population continues to employ plant-derived therapeutics [4]. This is due to the abundance of exceptionally useful and robust components found in plants called secondary metabolites [5].

Medicinal plants have been utilized as a source of medicine since ancient times. Medicinal herbs have been utilized for centuries to address health issues and mitigate the spread of infectious diseases. The exploration of novel medicines through the identification, isolation, and characterization of bioactive chemicals from plants has contributed to the advancement of pharmaceutical and health fields [6]. Phytochemicals, being a source of novel elements, play a key role in development of new novel drugs in pharmaceutical industries [7]. Like, over 60% of oncology chemotherapy medicines are plant-derived [8]. During the last 3 decades, multidrug resistance has emerged due to antimicrobial resistance as a result of the recurrent and continuous utilization of single drugs for similar therapeutic target. Due to the emergence of multidrug resistance, there is an ongoing imperative to investigate novel avenues for antibiotics to address the challenge of antimicrobial resistance [9].

Secondary metabolites derived from plants are produced during their life cycle as a result of metabolic processes [10, 11]. Traditional medicines based upon broad knowledge of herbal plants offer promising capacity to cope with multidrug resistance [12]. Herbal medications exhibit a diverse array of biological actions, making them effective in the management of various diseases [13]. The integration of medicinal and nutritional methods has the potential to serve as a powerful tool in managing a wide range of ailments [14]. As a result of plant metabolism, secondary metabolites are produced as intermediate or end products [15]. In plants, microorganisms, and herbivores, secondary metabolites serve as defense agents by blocking intracellular targets [16].

*Calotropis gigantea* (L.) R. Br. (Asclepiadaceae) belongs to the family of Apocynaceae, the well-known plant throughout the tropical world and native to the tropical and subtropical parts of Asia and tropical Africa. *Calotropis gigantea* (crown bloom) commonly known as milk weed grows in variety of soil and environment. *Calotropis* is native of India and distributed in several countries like Pakistan, Saudi Arabia, China, Sri Lanka, Thailand, Malaysia, Indonesia, Nepal, Brazil, Chile, Australia, Dominica, Colombia, and Morocco [17]. Literature has reported several phytochemical components in different parts of *Calotropis,* particularly leaves. Flower, bud, and roots of *Calotropis* contain alkaloids, carbohydrates, glycosides, flavonoids, sterol, saponins, phenolic compounds, tannins, acid compounds, peroxides, resins, polyuronides, amino acids, and proteins [18].

Various components of the plant possess significant therapeutic potential in treating diverse ailments. Literature reported several pharmacological and medicinal values of *Calotropis* like anticancer [19, 20], antimicrobial [21], antiviral [22], cytotoxic [23], fibrinolytic [24], CNS [25], antitumor and antidiarrheal [26], anthelmintic [27], anti-inflammatory [28], antidote [29], and wound healing [30] activities. Besides *Calotropis* medical values, its different parts have several uses. For example, entire plants have fungicidal and insecticidal properties and can be used as biogas and as a replacement for petroleum products [31] [32, 33], while leaves are used as substitute for paper and indicators of heavy metals [34]. Studies performed in past using different approaches in plants have also explicated the innumerable biological activities including antimicrobial [35–40], anti-inflammatory [41], antihyperglycemic [41], antiproliferative [40, 42, 43], oxidative stresses [44], antidiabetic [43, 45, 46], cytotoxicity [47], and antioxidant [39, 40, 48]. Several other approaches have been used to characterize and evaluate the biological activities of plants [35, 37–39, 42, 49, 50].

There is a scarcity of substantial literature and information about *C. gigantea*. Therefore, we hypothesized that, like potential medicinal *C. procera*, *C. gigantea* may have promising potential medicinal properties with valuable phytochemicals, antioxidants, and biological activities. Our study investigated biochemical evaluation of *C. gigantea* crude extract, qualitative analysis of antioxidants (AOX), hydrolytic enzymes, and free radical scavenging properties of isolated phytochemicals. The proposed study aims to evaluate the phytochemicals of *C. gigantea*, identify promising extracts, and investigate their biological and hemolytic activities. However, reports have revealed the possible therapeutic value of *C. gigantea*. However, no substantial research or studies on indigenously cultivated *C. gigantea* have been conducted in the Pakistan region. Therefore, we devised this study to investigate and exhume *C. gigantea* phytochemical profile, antioxidants, hydrolases, and hemolytic and antibacterial properties. This research will provide valuable insights, particularly in the pharmaceutical field, for the development of targeted antimicrobial medications against specific microorganisms.

## 2. Materials and Methods

### 2.1. Plant Sample Collection and Fraction (Extract) Preparation

*C. gigantea* mature plant fresh leaves were collected from local area of Jinnah Garden (latitude/longitude: 31° 33′ 13.42″ N/74° 19′ 52.69″ E), Lahore, Pakistan. The plant materials were taxonomically authenticated by Professor M.N. Shahid, Department of Botany, University of Education, Lahore. A voucher specimen for *C. gigantea* (No. 00C101, collection date: 15.01.2023) was deposited for reference in the Department of Botany, University of Education, Lahore. Leaves of the *C. gigantea* were washed with distilled water several times and further with deionized distilled water to remove any debris and impurities. Collected leaves were kept under shade for 15 days at room temperature (25°C) for drying. Phytochemical constituents were determined for *C. gigantea*. Leaves were grinded to fine pulverized powder using liquid nitrogen. 10 g powder was extracted with 100 mL ethanol solution (7:3 v/v ethanol: distilled water) for 50 min. Crude extract was then filtered using filter paper. The semisolid crude extract was subjected to concentrate using a rotary evaporator to obtain the plant extract in its crude form. The collected extract was subsequently kept at a temperature of 4°C until further use.

### 2.2. Extract Preparation (Maceration)

The *C. gigantea* leaf samples were dried for 7 days in an area free of direct sunlight. After drying, the leaves were ground into a fine powder. Prepare five containers, each corrected containing 200 g of *C. gigantea* powder, and macerate for 72 h (3 days) using 2000 mL of methanol, n-hexane, chloroform, ethyl acetate, and n-butanol separately. After maceration, the extract was filtered via a Buchner funnel and filter paper. The extract was concentrated by filtering it via a rotary evaporator before being utilized for phytochemical screening. The yield of each extract was estimated in relation to dried material. The extracts were kept cold and dry at 4°C until further use.

(1) $$\begin{array}{c} \%\text{ Extraction yield}=\frac{\text{mass of extract}}{\text{mass of dried}}\times 100\%. \end{array}$$

### 2.3. Phytochemicals (Flavonoids, Terpenoids, Saponins, Tannins, and Quinones)

Active metabolite constituents were identified using standard protocols. Flavonoids were determined following Madaan et al. [51]. Flavonoid contents were expressed as quercetin equivalents (mg quercetin/g sample) [52]. Terpenoids were estimated following Caceres et al. [53]. Saponins and tannins were determined by prescribed methodology of Sofowora [54] and Evans [55]. Glycosides were estimated following the Gul et al. [56] methodology. Quinone was measured following the Harbone [57] and Kokate [58] methodology.

### 2.4. Antioxidants (Superoxide Dismutase (SOD), Peroxidase (POD), and Catalase (CAT))

SOD activity was determined by measuring inhibition in photo reduction of nitro blue tetrazolium (NBT) by SOD enzyme Dixit et al. [59] method. Giannopolitis and Ries [60] methodology was followed by measuring the SOD ability to inhibit the photochemical reduction of NBT. The POD and CAT activity was determined following the method of Chance and Maehly [61] at wavelength of 470 and 240 nm, respectively, using a spectrophotometer (Varian, CARY-300 Bio Spectrophotometer, Conquer Scientific, Poway, CA).

### 2.5. Hydrolytic Enzymes (Protease (PROT) and Amylase (AMYL))

PROT activity was determined by the casein digestion approach reported by Drapeau [62], and absorbance of filtrate was measured at 280 nm wavelength. Enzyme activity was expressed on protein basis [63]. AMYL activity was performed according to the documented method of Bashary and Khatik [64]. The inhibition (%) of α-amylase was calculated using formula below.

(2) $$\begin{array}{c} \text{Amylase inhibition }\left(\%\right)=\frac{\text{Absorbance }\left(\text{control}\right)\;–\text{ Absorbance }\left(\text{sample}\right)}{\text{Absorbance }\left(\text{control}\right)}\times 100. \end{array}$$

### 2.6. Total Phenolic Contents (TPCs)

TPCs were estimated using colorimetric method as mentioned by Ainsworth and Gillespie [65] with little modifications. Extract was prepared (1 mg/mL) with individual solvents. 0.5 mL of Folin–Ciocalteu reagent was added into 0.5 mL of each extract, and the total volume was adjusted to 8.5 mL with distilled water. Prepared extracts were kept at 25°C for 10 min and then 20% sodium carbonate (1.5 mL) was added. Extracts were incubated for 20 min at 40°C. The developed blue color intensity absorbance was measured at 760 nm using UV-visible spectrophotometer.

### 2.7. Total Flavonoid Contents (TFCs)

TFCs were assessed following the method prescribed by Dewanto et al. [66]. Absorbance was measured spectrophotometrically at 510 nm. Samples measured in triplicates were calculated as mean values.

### 2.8. Free Radical Scavenging Activity (1,1-Diphenyl-2 Picrylhydrazyl (DPPH) Assay)

The DPPH was performed as described by Tepe et al. [67]. Aliquots of fractions were added to ethanol solution of DPPH. Absorbance of samples was measured at 516 nm and inhibition activity 50% was determined along with control (ascorbic acid). The DPPH activity (% inhibition activity) was measured using the following formula.

(3) $$\begin{array}{c} \%\text{ Inhibition activity }\left(\text{DPPH}\right)=\frac{\text{Control absorbance }–\text{ sample absorbance}}{\text{Control absorbance}}\times 100. \end{array}$$

### 2.9. Microorganisms (Bacterial and Fungal Strains)

Bacterial strains, such as *Escherichia coli* (ATCC 2592), *Pseudomonas aeruginosa* (ATCC 27853)*, Enterococcus faecalis* (ATCC 29212), and *Acinetobacter baumannii* (ATCC 19606), and fungal strains *Aspergillus fumigatus* (ATCC 204305), *Aspergillus flavus* (ATCC 9643), and *Aspergillus niger* (ATCC 1015) were used to assess the fractions' activity.

### 2.10. Disc Diffusion and Microdilution Broth Approach

Antimicrobial activity of *C. gigantea* leaves fractions was measured by disc diffusion method as described [68]. Ciprofloxacin and fluconazole (50–100 mg/mL) were used as positive control for bacteria and fungi, respectively. Antimicrobial activity was checked by measuring inhibition zone. Minimum inhibitory concentration (MIC) for fractions was determined following microdilution broth susceptibility method as reported [69]. A series of dilutions in the range of 20–100 mg/mL of extract in 96 well microplates including positive and negative control were added onto the microplates with 50 µL of tested solutions. A 50 μL of suspension of tested microbial strain with 5 × 10^5^ CFU/mL of standard microbial strain was inoculated on microplates at 37°C for 24 h for bacteria and at 30°C for 72 h for fungi. Following incubation, the plates were examined for a transition in color from yellow to red to purple, indicating the presence of live bacteria. The MIC was determined as the lowest concentration of plant extracts that did not result in any observable color change. The experiment was conducted three times. The antifungal activity values were calculated as the average of the zones of inhibition (mm) measured in triplicate for each extract treatment. The ZOI diameters (mm) were evaluated by comparing them with the fungicide fluconazole (positive control).

### 2.11. Hemolysis Assay

The cytotoxicity test was performed to determine the hemolytic activity in different fractions of *C. gigantea*. Plant extract of 1 mg/mL dissolved in DMSO (10%) was kept at room temperature for few minutes. Around 5CC fresh venous blood was collected from healthy humans and placed in heparinized tubes. Collected human venous blood was gently mixed and poured into 15 mL tube and centrifuged at room temperature (25°C) for 15 min at 850 × g. Hemolytic activity was measured as described by Yang et al. [70]. Absorbance of free erythrocytes was measured using UV-vis spectrophotometer at 540 nm. Each fraction was repeated thrice, and the average of triplicate values was measured.

### 2.12. Statistical Analysis

The experimental data were subjected to analysis of variance (ANOVA) to check the significance by using asstatistical program (SPSS 10.0). Each experiment was performed in triplicate. Significant differences among means were compared using Duncan multiple range test (DMR) at *p* < 0.05. Analysis for all parameters was performed in triplicates and expressed as mean ± S.E for all measurements.

## 3. Results

Phytochemical screening disclosed extracts encompassing alkaloids, saponins, tannins, terpenoids, glycosides, flavonoids, and quinones (Table 1). All extracts embodied alkaloids, saponins, and tannins, whereas the remaining compounds were found in only one or two extracts. The analysis of the methanolic extract revealed the presence of alkaloids, saponins, flavonoids, and terpenoids except quinones. On the other hand, the n-hexane extract included alkaloids, tannins, saponins, and quinones. All active metabolites except quinones were found in n-butanol extract, while chloroform contained all phytochemicals except glycosides (Table 1). Our study showed higher extract yield in n-butanol (5.05%), followed by methanol (4.82%), ethyl acetate (2.91%), chloroform (2.74%), and n-hexane (2.38%). Maximum extract yield was reported in n-butanol fraction while minimum was noticed in n-hexane fraction.

**Figure 1:**
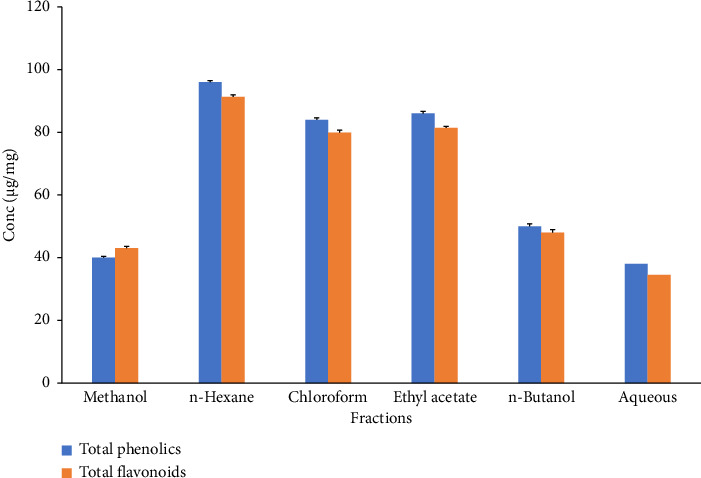
Total phenolics and total flavonoids of different extracts of *C. gigantea*.

**Figure 2:**
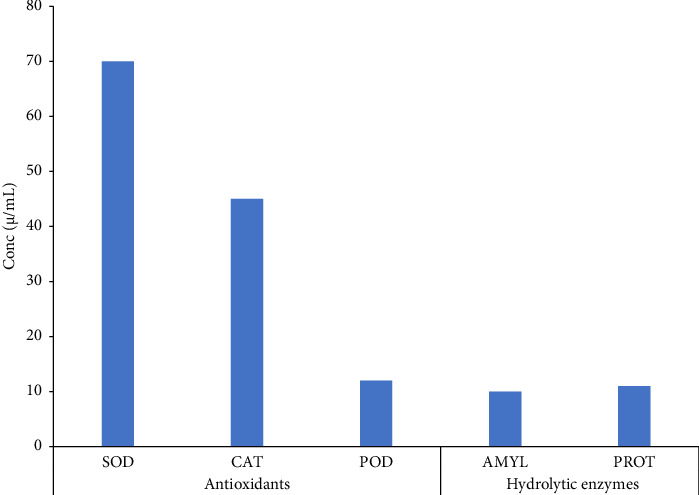
Antioxidant concentration of *C. gigantea* in ethanolic extract. Bars in the graph are pooled from three independent replicates (*n* = 3) and show the statistical difference (*p* < 0.05). SOD = superoxide dismutase; CAT = catalase; POD = peroxidase; AMYL = amylase; PROT = protease.

The phenolic contents in the extracts of *C. gigantea* were identified based on the polarity of the solvents employed. *n*-Hexane, chloroform, and aqueous fractions possessed different TPCs of 96 μg EAG/mg, 84 μg EAG/mg, and 86 μg EAG/mg, respectively (Figure 1). At the same time, *n*-butanol and methanol extract showed minimum phenolic contents of 50 μg EAG/mg and 40 μg EAG/mg, respectively (Figure 1). Our investigation revealed a similar quantitative pattern for TFCs. Maximum flavonoid levels were found in n-hexane, ethyl acetate, and chloroform (Figure 1). Significant differences have been noted between TPCs and TFCs (*p* < 0.05). Among antioxidants and hydrolytic enzyme activities, maximum activity was reported by SOD, followed by CAT, POD, AMYL, and PROT (Figure 2). Significant differences were noted for antioxidants and hydrolytic enzymes (*p* < 0.05).

The antioxidant capacity of *C. gigantea* was assessed by employing the DPPH assay, which evaluates its ability to scavenge free radicals. Methanolic, aqueous, n-hexane, and ethyl acetate fractions inhibited DPPH by 90%–99%, 88%–99%, 71%–97%, and 76%–97%, respectively, at doses ranging from 50 to 200 μg/mL (Table 2). The DPPH inhibition activity of chloroform and n-butanol varies between 45%–94% and 32%–85%, respectively, at different extract concentrations. All the fractions in our study showed maximum inhibition activity at the minimum concentration except the *n*-butanol fraction. We observed remarkably significant differences (*p* < 0.05) in DPPH activity among varying concentrations of *C. gigantea* fractions (Table 2).

**Figure 3:**
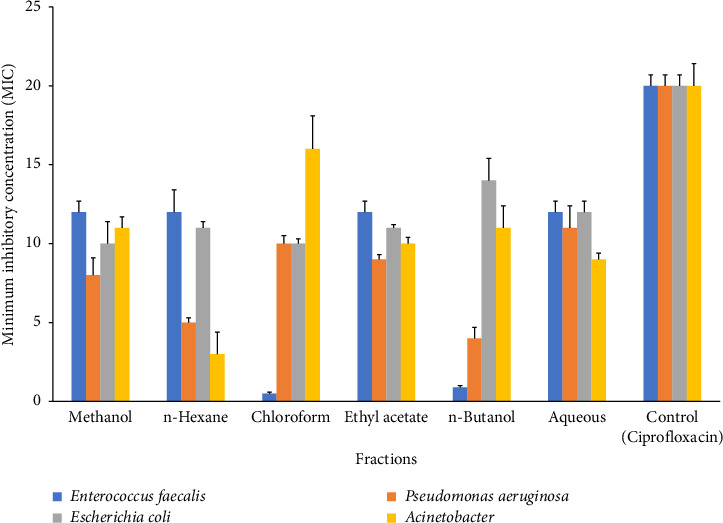
Inhibition zones in Gram-negative and Gram-positive bacterial strains formed by *C. gigantea* extracts with standard (ciprofloxacin). Bars in the graph are pooled from three independent replicates (*n* = 3) and show the statistical difference (*p* < 0.05).

Different types of bacteria and fungi were subjected to microbial activity testing, and the obtained data were subjected to statistical analysis. The findings of our investigation indicate that the plant extracts exhibit significant antibacterial properties against the Gram-positive bacteria strain (*E. faecalis*). The n-hexane extract exhibited the most pronounced inhibition zone activity, followed by methanol, aqueous, and ethyl acetate, all of which demonstrated comparable inhibition zone activity (Figure 3), which was lower than standards (ciprofloxacin 20 μg/mL). Among Gram-negative bacteria, *P. aeruginosa*, methanol and ethyl acetate showed almost similar results, while *n*-hexane and *n*-butanol documented the similar inhibition activity. Chloroform showed the maximum inhibitory effect against *P. aeruginosa* followed by ethyl acetate (Figure 3). The inhibitory activity in E. coli is ranked in increasing order as follows: methanol > chloroform > *n*-hexane > ethyl acetate > aqueous > *n*-butanol. The pattern of decreasing inhibitory activity was seen in *A. baumannii* as follows: *n*-hexane < aqueous < ethyl acetate < *n*-butanol < methanol < chloroform *A. baumannii* (Figure 3).

**Figure 4:**
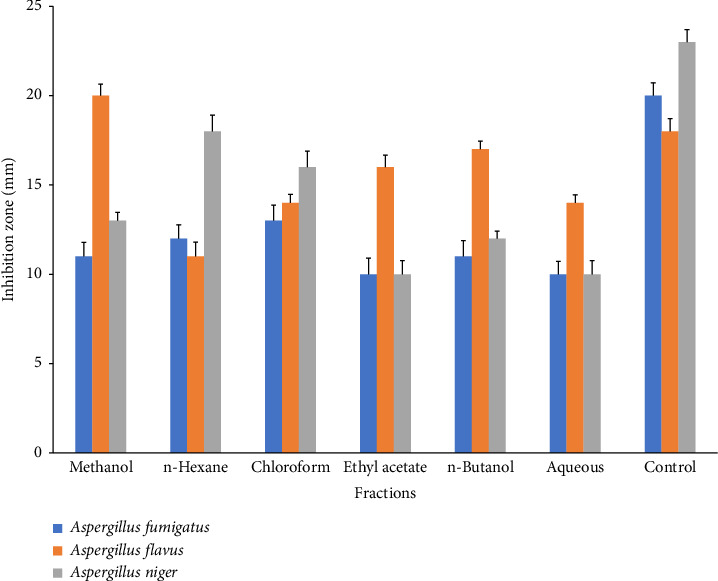
Inhibition zones in fungal strains formed by different *C. gigantea* fractions with control. Bars in the graph are pooled from three independent replicates (*n* = 3) and show the statistical difference (*p* < 0.05).

**Figure 5:**
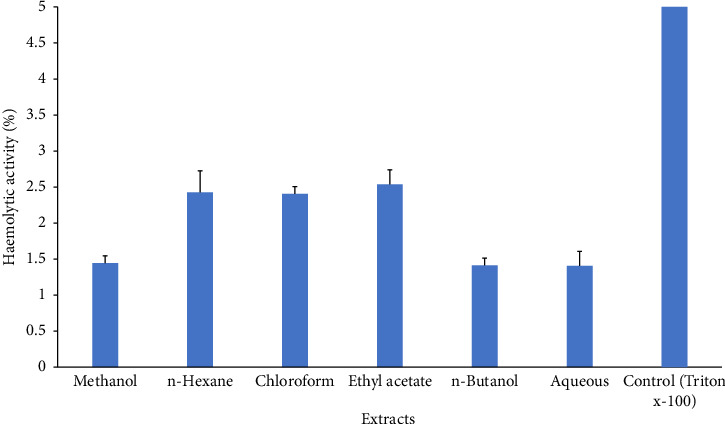
Hemolytic activity of different extracts of *C. gigantea.* Bars in the graph are pooled from three independent replicates (*n* = 3) and show the statistical difference (*p* < 0.05).

*C. gigantea* exhibits significant antifungal properties, as evidenced by the many extracts that demonstrate varying degrees of antifungal activity. Among extracts, chloroform extract showed the greatest inhibition activity against *A. fumigatus*. Methanol extract displayed maximum antifungal activity against *A. flavus* strain. *n-*Hexane exhibited greater inhibition potential against *A. niger* (Figure 4). *C. gigantea* extracts have shown promising hemolytic activity in our study. Increasing order profile of hemolytic activity, i.e., aqueous > *n*-butanol > methanol > *n*-hexane > chloroform > ethyl acetate, was noticed in our results (Figure 5).

## 4. Discussion

The phytochemical analysis of crude extracts derived from the leaves of *C. gigantea* disclosed the presence of alkaloids, saponins, tannins, terpenoids, cardiac glycosides, and terpenoids in n-butanol, chloroform, and methanol. The most reliable evidence for our study emanates from prior papers [71–74] which support and show *C*. *gigantea* as medicinal plant. However, quinones were found to be absent in the *n*-butanol extract. Saponins, flavonoids, terpenoids, and glycosides were not detected in *n*-hexane extract. Such profile was documented previously [75]. Higher amounts of TPC were obtained with hexane-based solvent followed by chloroform, ethyl acetate, *n-*butanol, methanol, and aqueous. The findings of the current studies align with previous research that supports the presence of TPC in various fractions. The TPC of *C. gigantea* in our findings aligns with the previously reported data[76], higher than that recorded earlier for the methanol extract of *Calotropis* [61]. The variation in our study's findings might be traced to various factors, such as the characteristics of the solvents used and the nature of the plant material extracted [77]. Elevated TFCs were obtained using hexane as solvent. Hexane proved to be highly effective solvent for the extraction of TFC from *C. gigantea*. The upsurge in TFC observed in various extracts in our tests can be ascribed to the enhanced antioxidant capacity of these extracts. The variation in TPC and TFC seen in our study can be attributed to the influence of growth conditions, plant tissues, and maturity at harvest [78]. Taking into account, heightened TPC and TFC from *C. gigantea* plant are due to its potential antioxidant properties [79].

Our studies elucidated higher SOD and CAT activities in *C. gigantea* as reported earlier [80, 81]. Higher activity of SOD can be attributed to its important role in dismutation of superoxide radicals. As far as we know, there is no available information on *Calotropis* addressing the estimation of PROT in various fractions. However, four cysteine proteases have been recognized in *C. gigantea* [82]. α-Amylase measured in our studies speculated that its presence in *C. gigantea* may be due to presence of phytochemicals such as tannins, saponins, and flavonoids in plants [83]. Stable DPPH method is used to measure the radical scavenging capacity in solvents of different polarity. The correlation between elevated phenolic concentrations or heightened hydroxylation levels of phenolic compounds and elevated DPPH activity, hence enhancing the antioxidant potential of a plant extract or related molecule, is widely acknowledged. Our studies elucidated that methanol and aqueous extract of *C. gigantea* showed maximum scavenging activity (% inhibition 99 at 50 μg/mL) followed by ethyl acetate and *n*-hexane (% inhibition 97 at 50 μg/mL) and chloroform and *n*-butanol (% inhibition 91 and 32, respectively, at 50 μg/mL). Reports also suggested the maximum radical scavenging activity of methanol in *Calotropis* [84]. Conversely, the extracts ethyl acetate, n-hexane, chloroform, and n-butanol exhibited lower DPPH activity compared to the other investigated extracts. This can be attributed to their reduced polarity [85] and the extent of hydroxylation of phenolic compounds.

The findings of this study indicate that all fractions had significant bactericidal efficacy against Gram-positive bacteria, except chloroform and n-butanol. Typically, fractions exhibit more activity on Gram-positive bacteria in comparison to Gram-negative bacterial strains. This phenomenon has been previously described and explained as variations in cell wall structure [86]. The ability of fractions to show bactericidal activity against different bacterial strains displayed the presence of hydrophobic and hydrophilic antibacterial compounds and their permeability [87]. The observed findings indicate that chloroform and n-butanol fractions did not exhibit a significant inhibitory effect on *E. faecalis*. Previous investigation has similarly documented comparable results for these fractions, indicating that aqueous and methanol solvents are more effective [88]. Gram-negative bacterial strains show a considerable variation in response to different fractions. Among Gram-negative bacterial strains, *P. aeruginosa* and *E. coli* showed almost similar inhibitory activity against chloroform, ethyl acetate, and aqueous fractions. The observed differences in bactericidal activity can be explained by the presence of distinct bioactive constituents and their variable levels of concentration across different fractions [89, 90]. Our findings highlighted that *A. baumannii* showed more sensitivity as compared to other Gram-negative bacterial strains. Similar results have been reported earlier [91] that can be explained as presence of variable bactericidal compounds in *Calotropis* [91].

Based on our research findings, it is apparent that various fractions of *C. gigantea* exhibited inhibitory effect against distinct fungal species. *A. fumigatus* exhibited reduced sensitivity in the methanol extract which is consistent with an earlier study [92, 93] as compared to *A. niger* and *A. flavus* which showed higher sensitivity in methanolic extraction which is in accordance with previous report [94]. *A. flavus* in our studies showed the maximum inhibitory activity against methanol, ethyl acetate, chloroform, and butanol fractions. Similar results were reported earlier [95]. The antifungal properties of phytochemicals can be attributed to their ability to disrupt the cytoplasmic membrane, induce cytoplasmic granulation, and modulate the activity of intra and extracellular enzymes through inhibition and activation [96]. These biological processes may occur individually or concurrently, resulting in mycelium growth inhibition. Proteolytic enzymes in fungal cell walls break down β 1,6 and β 1,3 glycans, as well as chitin polymer [96]. *C. gigantea* antifungal action is not well understood. In our findings, the aqueous fraction of *C. gigantea* displayed inhibitory efficacy on the tested fungal strains, which is remarkably similar to the prior studies [71]. The active components of *Calotropis* may exert their effects by the suppression of fungal cell wall formation, as well as through the synthesis of amino acids, proteins, and sphingolipids [97].

In our study, hemolytic activity of different fractions of *C. gigantea* was screened against normal human erythrocytes. *n*-Hexane, chloroform, and ethyl acetate showed similar hemolytic activity followed by methanol, *n*-butanol, and aqueous. In comparison to the control, all fractions exhibited reduced hemolytic activity toward human erythrocytes. The presence of saponins in plants accounts for the hemolytic activity [98]. Saponins have been found to enhance membrane permeability [99], thus rendering them significant adjuvants in this situation. However, they cause hemolysis because of similar property [98]. In our investigation, hemolytic activity varied among fractions; this variance can be attributed to a number of parameters, including saponin side chain, membrane composition, and environmental conditions [99]. Few plants have been studied for their hemolytic action [98, 99]. By comparing the results, we can conclude that *C. gigantea* hemolytic activity in human erythrocytes is not particularly noticeable, and so fraction use is safe.

## 5. Conclusion

*C. gigantea* has been used in traditional medicines due its pharmacological importance. Phytochemical constituents, antioxidant profile, and bioactive investigations of *C. gigantea* have not been studied exclusively yet. In our study, methanolic, aqueous, and ethyl acetate fractions revealed the maximum DPPH and antibacterial activity while chloroform extract showed promising antifungal activity. The bioactive constituents of this plant can be used as source of antioxidants in pharmaceuticals and food supplements. However, this report concluded that there should be standard suitable extraction process that can be employed to recover maximum quantity of potent antioxidants and phytochemical constituents from *C. gigantea* plant material.

### 5.1. Study Limitations, Challenges, and Recommendations

The study's limitations include an emphasis on a single geographic region (Central Punjab, Pakistan) for the collecting of *C. gigantea* leaves, which may limit the results' applicability to other regions. *C. gigantea* species, environmental conditions (soil, cultivation tactics, climate, harvesting, storage, processing, and handling), extraction (analytical) procedures, solvents, and other characteristics all have an impact on the findings, and hence these variables cannot be reliably standardized. Antimicrobial experiments with more bacterial and fungal species need to be conducted to evaluate against *C. gigantea* fractions. Other in-depth research employing other solvents and different plant tissues is needed to fully grasp its potential medicinal benefits. We also highly recommend the structural explication of *C. gigantea* bioactive compounds and their in silico studies against different diseases.

## Figures and Tables

**Table 1:** Phytochemical analysis of different extracts of *C. gigantea*.

Phytochemical tests	Phytochemicals					
	*n*-Butanol	Chloroform	Ethyl acetate	*n-*Hexane	Methanol	Aqueous
Alkaloids	+	+	+	+	+	+
Saponins	+	+	+	+	+	−
Tannins	+	+	+	+	+	+
Flavonoids	+	+	+	−	+	+
Terpenoids	+	+	−	−	+	+
Cardiac glycoside	+	−	−	−	+	−
Quinones	−	+	−	+	−	−

*Note:* + = present; − = absent.

**Table 2:** Radical scavenging activity (DPPH) in different fractions of *C. gigantea*.

Extracts	Concentrations (μg/mL)	Radical scavenging effect (%) (mean ± S.D)
Methanolic	200	90 ± 0.21^d^
	150	94 ± 0.30^b^
	100	92 ± 0.28^c^
	50	99 ± 0.09^a^

*n*-Hexane	200	97 ± 0.51^a^
	150	76 ± 0.42^b^
	100	71 ± 0.53^c^
	50	97 ± 0.16^a^

Chloroform	200	45 ± 0.38^d^
	150	81 ± 0.34^c^
	100	91 ± 0.22^b^
	50	94 ± 0.37^a^

Ethyl acetate	200	82 ± 0.19^c^
	150	93 ± 0.51^b^
	100	76 ± 0.58^d^
	50	97 ± 0.44^a^

*n*-Butanol	200	43 ± 0.19^c^
	150	60 ± 0.47^b^
	100	85 ± 0.24^a^
	50	32 ± 0.23^d^

Aqueous	200	97 ± 0.19^b^
	150	94 ± 0.18^c^
	100	88 ± 0.16^d^
	50	99 ± 0.53^a^

Ascorbic acid (positive control)	200	99 ± 0.05^a^
	150	98 ± 0.08^b^
	100	96 ± 0.11^c^
	50	94 ± 0.09^d^

*Note:* Superscript letters show the statistically significant differences.

## Data Availability

All the data related to the manuscript have been mentioned.
